# Multiple Data Demonstrate That Bacteria Regulating Reproduction Could Be Not the Cause for the Thelytoky of *Diglyphus* *wani* (Hymenoptera: Eulophidae)

**DOI:** 10.3390/insects13010009

**Published:** 2021-12-22

**Authors:** Sujie Du, Fuyu Ye, Qijing Wang, Yongxuan Liang, Weijie Wan, Jianyang Guo, Wanxue Liu

**Affiliations:** 1 State Key Laboratory for Biology of Plant Diseases and Insect Pests, Institute of Plant Protection, Chinese Academy of Agricultural Sciences, Beijing 100193, China; dsujie@126.com (S.D.); fuyuyeHNDX@163.com (F.Y.); qijingwong@163.com (Q.W.); aaliangyongxuan@163.com (Y.L.); 82101202331@caas.cn (W.W.); 2 Institute of Entomological Science, College of Agriculture, Yangtze University, Jingzhou 434025, China; 3 Department of Life Sciences, Hunan Normal University, Changsha 410081, China

**Keywords:** reproductive mode, Chalcidoidea, endosymbionts, PCR detection, 16S rRNA gene

## Abstract

**Simple Summary:**

In Hymenoptera, some species have two reproduction modes, that is, arrhenotokous and thelytokous parthenogenesis. Although arrhenotoky is common, the thelytoky is not rare. Thelytoky in Hymenopteran species has several causes and is generally induced by bacteria. Here, we examined a thelytokous Chalcidoidea parasitoid, *Diglyphus* *wani*, which was recently reported to be a dominant parasitoid attacking agromyzid leafminers. In this study, we aimed to combine multiple methods to explore whether endosymbionts induce thelytokous *D.* *wani*. Our findings revealed that the thelytoky of *D*. *wani* was not to be associated with the actual presence of the endosymbionts. Thus, *Diglyphus* *wani* is the second reported eulophid parasitoid for which thelytoky is not linked with the presence of endosymbionts.

**Abstract:**

In Hymenoptera parasitoids, the reproductive mode is arrhenotoky, while a few species reproduce by thelytoky. The thelytoky of Hymenoptera parasitoids is generally genetically determined by the parasitoids themselves or induced by bacteria, including *Wolbachia*, *Cardinium*, and *Rickettsia*. *Diglyphus* *wani* (Hymenoptera: Eulophidae), a recently reported thelytokous species is a main parasitoid attacking agromyzid leafminers. To assess whether endosymbionts induce thelytoky in *D.* *wani*, we performed universal PCR detection and sequenced the V3-V4 region of 16S ribosomal RNA gene. In addition, bacteria were removed through high-temperature and antibiotic treatments, and the localized bacteria were detected using FISH. Based on general PCR detection, *Wolbachia*, *Cardinium*, *Rickettsia*, *Arsenophonus*, *Spiroplasma*, and Microsporidia were absent in laboratory and field individuals of thelytokous *D.* *wani*. Furthermore, 16S rRNA gene sequencing revealed that the dominant endosymbionts in thelytokous *D.* *wani* were not reproductive manipulators. High-temperature and antibiotic treatment for five consecutive generations cannot reverse the thelytokous pattern of *D.* *wani*, and no male offspring were produced. Moreover, no bacterial spots were found in the ovaries of *D.* *wani*. Thus, it is considered that the thelytoky of *D*. *wani* does not result in the presence of endosymbionts. This species is thus the second reported eulophid parasitoid whose thelytoky appears not to be associated with endosymbionts.

## 1. Introduction

Hymenopteran parasitoids are important natural antagonists that attack phytophagous pests in agroforestry ecosystems [1,2,3,4,5]. Sex determination in Hymenoptera parasitoids is generally a haplodiploid system [6], and the majority of the parasitoids are arrhenotokous species (haploid males develop from unfertilized eggs, and diploid females develop from fertilized eggs). The reproductive mode of a few species is thelytoky (diploid females develop from unfertilized eggs); however, reproduction is generally not cyclical thelytoky [7,8,9], where arrhenotokous and thelytokous alternately occur in the life cycle [10], but is generally due to genetic background or microbial infection [7,11,12].

Thelytoky of Hymenopterans is always associated with and induced by microorganisms [13,14]. At least three genera of endosymbionts, including *Wolbachia*, *Cardinium*, and *Rickettsia*, could induce thelytoky [7,15,16]. These endosymbionts are usually removed or reduced to produce males via high temperatures or antibiotics [8,11,17,18,19,20]. Thelytoky could also be genetically determined; however, only several parasitoids of Ichneumonoidea, Braconidae, Trichogrammatidae, and Aphelinidae in Hymenoptera were discovered to be genetically thelytokous [12,18,21,22].

**Figure 1:**
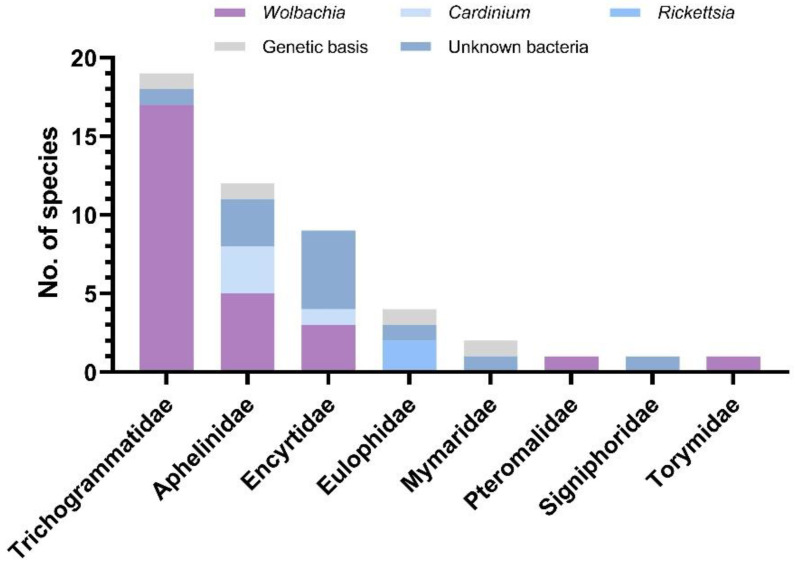
Endosymbiont infection and relevant causes of thelytoky in Chalcidoidea species based on our statistics and of van der Kooi et al. [14].

At present, at least 236 Hymenoptera: Chalcidoidea thelytokous species have been reported based on our statistics and mainly from van der Kooi et al. [14]. Of these, endosymbiont infections, mainly caused by *Wolbachia*, *Cardinium*, and *Rickettsia* were observed in 70 species. *Wolbachia* is the most widespread in Chalcidoidea species, infecting eight families, especially Trichogrammatidae. However, many studies have solely detected *Wolbachia*, without considering other endosymbionts as the agents that induce thelytoky [13,14]. However, *Wolbachia*-induced thelytoky among confirmed species may be overestimated [13,14]. A total of 49 thelytokous Chalcidoidea species have identified the causes of thelytoky based on our statistics and partial data of van der Kooi et al. [14]. To visualize the causes of thelytoky for these 49 species, we plotted Figure 1. Only 45 of 49 thelytokous Chalcidoidea species were verified to be bacterial infections using high-temperature or antibiotic treatments, while four thelytokous species have a genetic basis (Figure 1). In addition, as shown in Figure 1, *Rickettsia* is present only in two Eulophidae species [8,16], and *Cardinium* is present only in Aphelinidae [15,18,23] and Encyrtidae of Chalcidoidea [24]. In summary, the reproductive inducement of 187 of 236 thelytokous Chalcidoidea species are yet to be revealed or are only described as bacterial-induced without further verification (i.e., high temperature or antibiotic treatment). Therefore, further research is necessary to explore the mechanisms underlying thelytoky in Chalcidoidea species for better application in the future.

Here, we present a study on a thelytokous parasitoid, *Diglyphus wani* (Hymenoptera: Chalcidoidea: Eulophidae) [9]. This species also has an arrhenotokous strain, and two strains (thelytokous and arrhenotokous *D*. *wani*) are the dominant parasitoids in agromyzid leafminers; both strains exhibit three host-killing characteristics (host-feeding, parasitism, and host-stinging), especially to native *Chromatomyia horticola* and invasive *Liriomyza huidobrensis* and *L. sativae* [9,25]. Moreover, we aimed to determine whether bacteria are involved in the thelytokous reproduction of *D. wani*. We performed a combination of methods: (1) PCR detection using universal and specific primers for known sex-manipulating endosymbionts, (2) antibiotic and high-temperature treatments to reverse sexual reproduction and produce males, (3) fluorescence in situ hybridization (FISH) using universal 16S rRNA gene probes to locate bacterial distribution in the ovary, and (4) high-throughput 16S rRNA gene sequencing. Understanding the role of sex-manipulating factors will allow us to explore ecological strategies for the successful evolution of thelytokous *D. wani*.

## 2. Materials and Methods

### 2.1. Laboratory Population

Thelytokous *D*. *wani* populations were collected in Xining, Qinghai, China (36°39′ N, 101°45′ E) in 2015 and arrhenotokous *D*. *wani* populations in Kunming, Yunnan, China (24°53′ N, 102°47′ E). To date, thelytokous *D*. *wani* has been reared for more than five years, and no male offspring have been obtained. Thelytokous *Neochrysocharis formosa* and *L. sativae* were collected in Beijing, China (40°39′ N, 116°16′ E) in 2013. Leaves of *Phaseolus vulgaris* containing the larvae of *L. sativae* from the second to the third instar were provided as hosts for the parasitoids in the laboratory. Both the leafminer and parasitoid colonies were reared under a 14 L:10 D photoperiod and 25 ± 1 °C.

### 2.2. Field Population

Thelytokous *D. wani* individuals were collected from plant leaves with parasitized leafminers in the field. The leaves were then placed in nylon gauze incubated at 25 ± 1 °C, 50 ± 10% relative humidity, and a 14 L:10 D photoperiod until adults emerged. Soon after parasitoids emerged, they were collected and frozen faintly at −20 °C. Each sample was placed in plastic pipes containing 99% ethanol for morphological identification. For general PCR detection, laboratory and field colonies of the thelytokous *D*. *wani* were used (Table 1). To ensure that there were no problems in general PCR detection, we utilized insects containing reproduction-regulated endosymbionts as positive controls (Table 1).

### 2.3. DNA Extraction

All specimens were sterilized with cold 75% ethanol for 5 min and washed five times with sterile 1× PBS (phosphate buffer saline; pH 7.4), following the procedure described by Guo et al. [26]. The different developmental stages of *D. wani* were collected from laboratory colonies, as follows: five single whole wasp adults and pupae, five pooled whole wasps, and 23 pooled eggs of 10 different wasps, five pairs of wasp ovaries dissected from 10 females under sterile conditions in 1× PBS, and five pairs of 3-day-old larvae. The DNA of the field specimens was extracted. All species were identified based on their morphology and the COI gene before further bacterial detection. All extracted DNA were used to perform general PCR detection.

To identify the bacterial community, we collected black pupae before parasitoids emerged. The surfaces of the pupae were cleaned three times with 75% alcohol and sterile ddH_2_O. Then, these pupae were transferred into 1.5 ml sterilizing centrifugal tubes. Each tube was placed in a single black pupa. Three biological replicates were set, and each replicate included approximately 30 individuals. DNA was extracted from all specimens using the QIAGEN blood or tissue genome kit (Hilden, Germany), following the manufacturer’s standard protocol. 

### 2.4. General Polymerase Chain Reaction (PCR) Detection

PCR detection of endosymbionts was performed using the specific 16S rRNA gene primers for *Rickettsia, Spiroplasma, Arsenophonus*, and Microsporidia, as reported by Foray et al. [27] (Table 2). *Wolbachia* was also assessed by targeting its surface protein (WSP) and 16S rRNA gene using the primer set 81F and 691R [28] and primer set 99F and 994R [29], respectively (Table 2). *Cardinium* was also detected using the primers reported by Weeks et al. [30] (Table 2).

The following known reproductive manipulators were used as positive controls: *Wolbachia* from *Encarsia formosa*, *Cardinium* and *Arsenophonus* from *Bemisia tabaci*, *Rickettsia* from *Neochrysocharis formosa*, *Spiroplasma* from *Tetranychus truncates*, and Microsporidia from *Plutella xylostella*. The PCR mix consisted of 0.4 μL Taq enzyme (2.5 U · μL^−1^), 0.4 μL dNTP (2.5 mM), 2.5 μL 10× buffer (containing Mg^2+^), 0.4 μL forward primer, 0.4 μL reverse primer, 50 ng genome template, and ddH_2_O was added to a final volume of 25 μL. The PCR conditions were as follows: the annealing temperatures varied at 48–60 °C, and the rest of the conditions were set uniformly: initial denaturation 95 °C for 3 min, followed by 35 cycles of denaturation at 95 °C for 20 s, annealing for 20 s, and extension at 72 °C for 60 s, and a single cycle of final extension 72 °C for 5 min. PCR amplification was performed using an ABI 9902 Thermal Cycler (Applied Biosystems, Singapore). 

After the PCR reaction, 4 µL of the PCR product was mixed with 0.4 µL of 10× loading buffer and then electrophoresed in 1% agarose solution (0.6 g agarose · 60 mL 1 × TAE) containing GelStain (TransGen Biotech, Beijing, China) at a voltage of 180 V and current of 400 mA for 15 min. After electrophoresis, bands were observed using a gel imaging system. Unpurified PCR products containing the targeted bands were directly sequenced by Tsing Ke Biological Technology (Beijing, China). All sequences were analyzed using the Basic Local Alignment Search Tool (BLAST) for homology search in the NCBI database.

### 2.5. Bacterial Community Diversity

We analyzed the bacterial communities of the pooled ~30 adults of thelytokous *D. wani*. Moreover, we performed a comparative analysis to address differences in bacterial compositions between the two strains of *D. wani* and thelytokous *N. formosa* as common hosts. In addition, ~30 larvae from the second to third instar of L. sativae were used for the same analysis. All specimens were obtained from the laboratory colonies. Each species had three biological replicates. The V3-V4 hypervariable region of the 16S rRNA gene was amplified using the forward primer 338F (5′-ACTCCTACGGGAGGCAGCA-3′) and the reverse primer 806R (5′-GGACTACHVGGGTWTCTAAT-3′), as described previously [40]. This region of the 16S rRNA gene was sequenced and analyzed by the Biomarker Technologies Company (Beijing, China). Sequences were trimmed, to exclude primer sequences using FLASH V1.2.7 [41]. Low-quality tags were further filtered using the Trimmomatic v0.33 [42]. The sequences were clustered into operational taxonomic units (OTUs) with a 97% similarity cutoff [43]. A Venn diagram was created to analyze similarities and differences between parasitoids and their host, *L. sativae*, using the effective sequence tags in R software [44]. The relationship between bacterial communities among groups was analyzed using the non-metric multidimensional scaling (NMDS) method based on OTU numbers and Bray–Curtis distance. When the stress was less than 0.2, the NMDS analysis was relatively reliable. These analyses were performed using the Biomarker Biocloud Platform (http://en.biocloud.net/private-cloud accessed on 10 November 2021). 

### 2.6. Antibiotic and High-Temperature Treatments

To test whether the thelytokous reproduction of *D. wani* could be reverted to arrhenotokous reproduction, antibiotic and high-temperature treatments were performed. If male adults could be produced using these treatments, it would be evidence of thelytoky induction. Briefly, parasitoids at the pupal stage were separated from the hosts to collect the emerged adults. One-day-old female were obtained and prepared for further tests. Ten newly emerged adult females were individually fed honey containing rifampicin (20 mg·mL^−1^) [8] or tetracycline (20 mg·mL^−1^) [17] for 24 h in a round glass petri dish, and then the 2nd to the 3rd instar of *L. sativae* were provided as hosts. Hosts and honey streaks were replaced daily until the death of the parasitoid. Antibiotic treatment was performed at 25 °C, while the high-temperature treatment was conducted at 34 °C initially in 20 females. Emerged females were fed with honey and stored at 25 °C in the same Petri dish to allow parasites for 24 h. Similarly, the hosts were grown under the same conditions. When black pupae begin to appear, the Petri dish was placed at 25 °C for emergence, as parasitoids may not emerge successfully at high temperatures. The experiment was performed for five generations using 20 first-generation females, and the sex was examined in each generation.

### 2.7. Fluorescence Microscopy

The endosymbionts that induce thelytoky can be inherited maternally [11]. Thus, endosymbionts can inevitably infect their reproductive systems [8,11]. Thus, we observed the localization of bacteria in the host’s reproductive tissues using FISH, which is a more sensitive method to detect the presence of bacteria. The universal bacterial probe EUB338 (5′-Cy5-GCTGCCTCCCGTAGGAGT-3′) targeting the 16S rRNA gene of bacteria was used [45]. Bacterial localization was performed by slightly modifying the FISH methodology described by Giorgini et al. [8]. Briefly, ovaries were extracted from adult bodies and fixed in 4% paraformaldehyde (PFA). After the fixation process, ovaries were hybridized in a buffer containing 10 pmol · mL^−1^ of EUB338-Cy5 fluorescent probe and observed under a Zeiss 980 laser confocal microscope. The specificity of the observed signals was verified using no-probe and RNase-digested controls. The nuclei of the ovarian cells were stained using 0.2 g · mL^−1^ 4′,6-diamidino-2-phenylindole (DAPI). To avoid technological problems, *N. formosa* infected by *Rickettsia* was used as a positive control.

## 3. Results

### 3.1. General PCR Screening

All 111 individual thelytokous *D. wani* collected from five different populations and examined in the laboratory and field were negative for *Wolbachia, Cardinium, Rickettsia, Arsenophonus, Spiroplasma*, and Microsporidia using general PCR detection with universal and specific primers. Highly sensitive PCR methods and testing of DNA samples extracted from single and pooled individuals of different developmental stages did not provide any evidence of endosymbionts infection. 

### 3.2. Bacterial Community Composition

**Figure 2:**
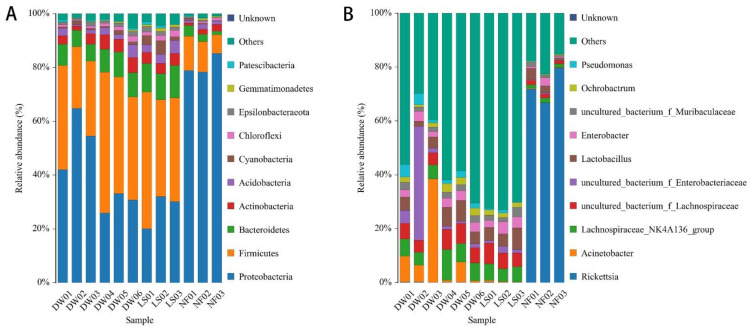
Relative abundance of the top 10 (**A**) phyla and (**B**) genera. The histogram represents the structure and diversity of the microbial community in different parasitoids and their host. The *x*-axes denote the samples, as follows: DW01~DW03 of arrhenotokous *Diglyphus wani*, DW04~DW06 of thelytokous *Diglyphus wani*, LS01~LS03 of *Liriomyza sativae*, and NF01~NF03 of thelytokous *Neochrysocharis formosa*. The y-axes denote the relative abundance (%) of the taxa annotated in the parasitoids.

Parasitoids and their hosts showed different bacterial communities (Figure 2A,B). At the phylum level (Figure 2A), Proteobacteria and Firmicutes were dominant in the thelytokous *D. wani*, arrhenotokous *D. wani*, and *L. sativae*, while Proteobacteria was dominant in the thelytokous *N. formosa*. At the genus level (Figure 2B), thelytokous D. wani and L. sativae shared similar dominant bacteria, namely Lachnospiraceae_NK4A136_group, uncultured_bacterium_f_Lachnospiraceae, and *Lactobacillus*. However, in arrhenotokous *D. wani*, Acinetobacter (18.06%) and uncultured_bacterium_f_Enterobacteriaceae (16.21%) were the dominant genera. *Rickettsia* was the most dominant bacteria in the thelytokous *N. formosa*, with a relative abundance of 67–80%. Contrarily, we detected a relatively low abundance of *Rickettsia* (0.1%) in two strains of *D. wani* and *L. sativae*. 

**Figure 3:**
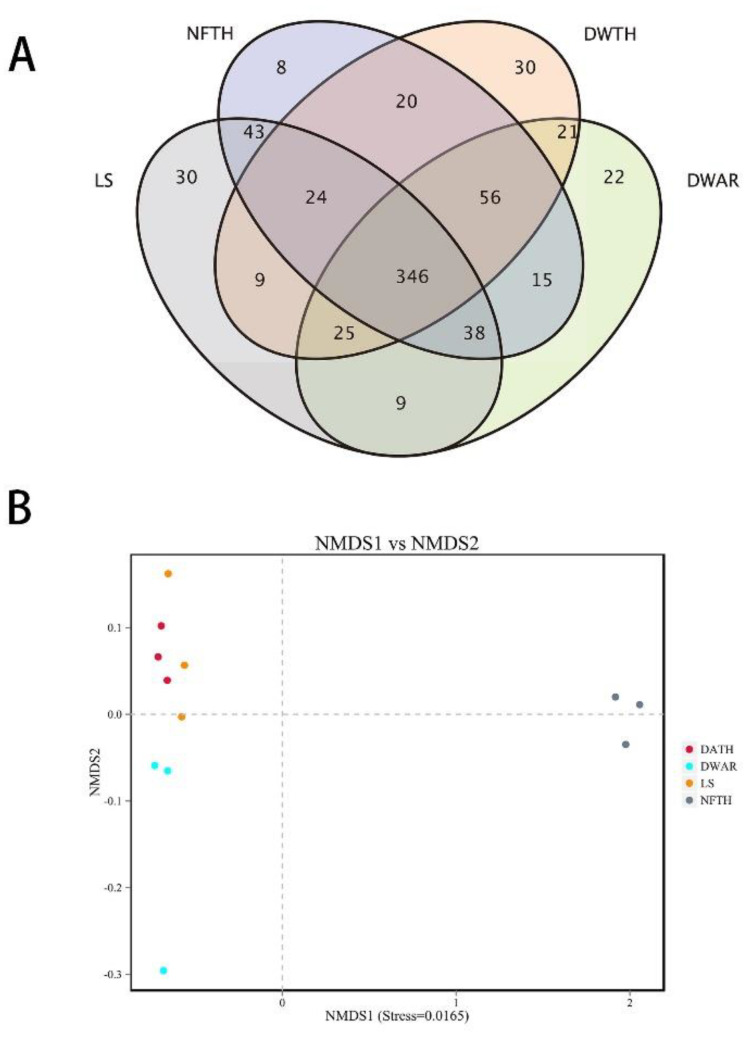
(**A**) Venn diagram showing the number of shared and unique genera in different parasitoids and their host. (**B**) Non-metric multidimensional scaling analysis based on the Bray–Curtis distance. DWAR, DWTH, LS, and NFTH represent arrhenotokous *Diglyphus wani*, thelytokous *Diglyphus wani*, *Liriomyza sativae*, and thelytokous *Neochrysocharis formosa,* respectively.

A Venn diagram reflecting the number of shared and unique genera from the samples showed that there were 346 genera common to the parasitoids and leafminers (Figure 3A). Although they share the same host, *L. sativae*, unique bacteria have been observed in different parasitoids. There were 30 genera unique to thelytokous *D. wani*, 22 genera unique to arrhenotokous *D. wani*, and eight species unique to thelytokous *N. formosa* (Figure 3A). However, the unique bacteria had relatively low abundance in all samples, including thelytokous *D. wani*. 

NMDS analysis demonstrated that there were significant differences among the groups (*p* = 0.001 < 0.01) (Figure 3B). A high similarity of bacterial distribution between the two strains of *D. wani* and *L. sativae* was observed, while the similarity between thelytokous *D. wani* and *L. sativae* was higher. In contrast, thelytokous *N. formosa*, dominated by *Rickettsia*, was clustered separately from the other groups (Figure 3B).

### 3.3. Effects of Antibiotic and High-Temperature Treatments

All progeny were females, and no males were produced under the five generations treated by the two different antibiotics, tetracycline and rifampicin, and high temperature (Table 3). 

### 3.4. Fluorescence Microscopy

**Figure 4:**
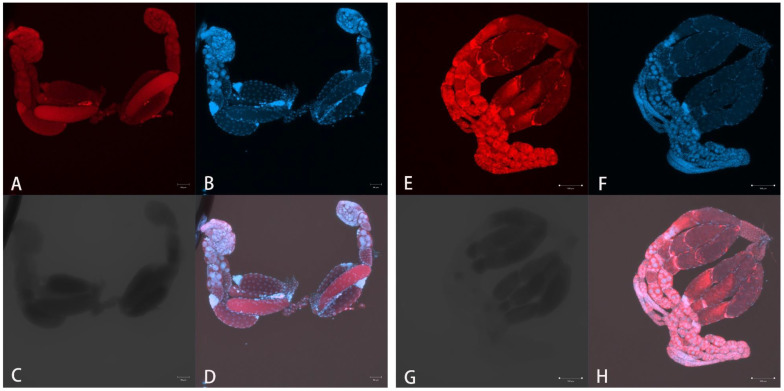
Fluorescence in situ hybridization (FISH) examination of adult ovaries. (**A**–**D**) *Diglyphus wani*, (**E**–**H**) *Neochrysocharis formosa*. Scale bars represent 50 µm. Ovaries are stained with (**A**,**E**) the universal bacterial probe EUB338-Cy3 and (**B**,**F**) DAPI. (**C**,**G**) Ovaries observed under bright field, (**D**) and (**H**) were merged with different channels. Scale bars in (**E**–**H**) represent 100 µm.

Ovaries of *D. wani* analyzed using FISH did not display any dense clusters or spots attributed to the presence of bacteria (Figure 4A–D). However, the ovaries of *N*. *formosa* displayed dense bacterial spots, revealing the presence of bacteria (Figure 4E–H), when processing and setting the same treatment and camera parameters as *D. wani* simultaneously.

## 4. Discussion

Microorganisms are the main reproductive manipulators in the thelytoky of Hymenoptera parasitoids including *Wolbachia*, *Cardinium*, and *Rickettsia* [7,15,16]. However, not all thelytokous parasitoids are induced by endosymbionts. In the current study, observations from thelytokous *D*. *wani* from field or laboratory populations suggested the exclusion of bacterial inducement for several reasons: (1) thelytoky seems to be not be revertible by antibiotics or heat high-temperature treatments; (2) no endosymbiont capable of inducing thelytoquia was detected either by PCR screening or by FISH; (3) their absence indicates that endosymbionts were not responsible for the present thelytoky in that species. Based on the results, *Rickettsia,* which is relevant to reproduction, was predominant among all bacteria in the thelytokous *N*. *formosa*. Similarly, *Rickettsia* was the dominant bacteria (93.67%) in thelytokous *Leptocybe invasa* (Hymenoptera: Eulophidae) [26]. In the thelytokous *D*. *wani*, although we also found *Rickettsia*, its abundance was extremely low (0.09%). Furthermore, a low bacterial titer could not maintain a stable thelytoky to produce males for parasitoids induced by endosymbionts [8,46,47]. In addition, other reproductive manipulators that have been reported, such as *Wolbachia* and *Cardinium*, were not discovered in the thelytokous *D*. *wani*. In our study, the predominant bacteria in the thelytokous *D*. *wani* were Lachnospiraceae_NK4A136_group, uncultured_bacterium_f_Lachnospiraceae, and *Lactobacillus*. However, these endosymbionts have not been reported to induce thelytoky in insects. Lachnospiraceae was assumed to store nutrients in the gut of insects [48] or break down plant residues [49]. Meanwhile, *Lactobacillus* was previously discovered in the thelytokous wasp *Leptocybe invasa* (Hymenoptera: Eulophidae) [26] and was associated with immunization [26], insect resistance [50], and positively affected the health of the hosts [51]. At the same time, we also examined the function of the different bacteria between parasitoids and their host. Unfortunately, differential bacteria was not reported to be involved in the thelytoky of species. Our results showed that *D. wani* did not conform to the main trend, where in thelytoky was induced by the three bacterial genera *Wolbachia*, *Cardinium*, and *Rickettsia*. To the best of our knowledge, *Thripoctenus javae* (Hymenoptera: Eulophidae) was the first reported species that do not have a verified endosymbiont that promotes thelytoky [52]. Thus, our study presented the second Chalcidoidea parasitoid (*D. wani*) in which thelytoky could be not associated with endosymbionts. 

Endosymbionts can be transmitted horizontally in different hosts [52,53]. Agromyzid leafminers, including *Liriomyza* species, are common hosts of *D. wani*. It has been previously reported that *Liriomyza trifolii* and *L*. *bryoniae* are occasionally infected by *Wolbachia* [54]. In addition, we also detected five *Liriomyza* species (*L*. *sativae*, *L*. *trifolii*, *L*. *bryoniae*, *L*. *huidobrensis*, and *L*. *chinensis*) and a species of *C*. *horticola* from China (unpublished data). However, *Wolbachia* was detected only in *L*. *bryoniae*, *L*. *huidobrensis*, and *L*. *chinensis* (unpublished data). In addition, *D. wani* infested from these three *Liriomyza* species was not involved in the regulation of reproduction using PCR screening (unpublished data). It is possible that this was due to limited bacterial transmission efficiency among different species or a strong immune system resistance of parasitoids. 

Notably, we did not find thelytokous *D. wani* males in either the laboratory colonies reared for 5 years or the field colonies. Thus, several possible hypotheses for the thelytoky of *D. wani* have been proposed, with each hypothesis leading to a non-reversible thelytoky. First, the genetic basis which was a single locus or gene would cause the thelytoky of *D*. *wani* [55,56,57]. In general, single-locus sex determination is not compatible with the formation of homozygous diploid females whose thelytoky is induced by endosymbionts infection and is usually found in species with genetically induced thelytoky, especially eusocial Hymenoptera [58,59]. In Hymenoptera parasitoids, only one species, *Lysiphlebus fabarum* (Hymenoptera: Braconidae), has been reported to be controlled by a single recessive locus [56]. Moreover, *Lysiphlebus fabarum* was found to be the genetic basis for thelytoky [60]. The thelytoky in *D. wani* could have originated from interspecific hybridization. In Chalcidoidea, the hybrid origin of thelytoky was only found in *T*. *cacoeciae* [12,61]. The lack of recombination between the parental genomes can fix heterozygous sites and allow the maintenance of the thelytoky of *T*. *cacoeciae* [12]. Furthermore, certain types of insect virus may mediate sex-ratio manipulation [62,63,64]. However, to the best of our knowledge, virus-induced thelytoky of parasitoids and other organisms has not been reported. With a long-term coevolution and interaction, the genes of the endosymbiont for thelytoky were partially inserted into its host genome [65,66,67,68,69]. In recent years, reports of horizontal gene transfer (HGT) from bacteria to their hosts have been increasing [70]. Nevertheless, the mechanisms by which these HGTs integrate into the host are not clear. Thus, the most likely trigger for the thelytokous of *D. wani* may be genetically determined. 

In addition, the cytogenetic mechanism of thelytokous *D. wani* could be explored. The mechanisms of thelytoky for restoring diploids were generally through central fusion, terminal fusion, gamete duplication or apomixis to restore diploidy [58,71,72]. Of these, gamete duplication was the most common cytological mechanism in Hymenopteran thelytokous parasitoids, especially species whose thelytoky was induced by *Wolbachia*. The cytogenetic mechanisms of some Hymenoptera parasitoids, for instance, *Encarsia guadeloupae* (central fusion), *Lysiphlebus fabarum* (central fusion), and *Meteorus pulchricornis* (apomixis), have been revealed [18,60]. Finally, our findings provide a theoretical foundation for exploring the causes and genetic mechanisms of the thelytoky of *D*. *wani*. Further studies will focus on two aspects of this: revealing genetic factors and the cytogenetic mechanism underlying thelytoky.

## Figures and Tables

**Table 1:** Thelytokous *Diglyphus wani* and species infected with microorganisms collected in this study.

Species	Reproductive Regulators (Microorganism)	Individuals	Province/Autonomous Region/Municipality	City	Sex	Individuals	Year	Host	Sampled Plant
*Diglyphus wani*	None	Laboratory	Beijing	Beijing	Female	45	2020	*Liriomyza sativae*	*Phaseolus vulgaris*
*Diglyphus wani*	None	Field	Qinghai	Xining	Female	19	2018	*Chromatomyia horticola*	*Pisum sativum*
*Diglyphus wani*	None	Field	Tibet	Lhasa	Female	14	2018	*Liriomyza huidobrensis*	*Sonchus oleraceus*
*Diglyphus wani*	None	Field	Hebei	Shijiazhuang	Female	13	2018	*Chromatomyia horticola*	*Brassica napus*
*Diglyphus wani*	None	Field	Beijing	Beijing	Female	20	2017	*Chromatomyia horticola*	*Brassica pekinensis*
*Encarsia formosa*	*Wolbachia*	Field	Hebei	Langfang	Female	5	2017	*Bemisia tabaci*	*Solanum lycopersicum*
*Bemisia tabaci*	*Cardinium*	Field	Xinjiang	Hami	Female	8	2017	-	*Solanum lycopersicum*
*Bemisia tabaci*	*Arsenophonus*	Field	Gansu	Tianshui	Female	10	2019	-	*Cucumis sativus*
*Neochrysocharis formosa*	*Rickettsia*	Field	Beijing	Beijing	Female	5	2017	*Liriomyza sativae*	*Vigna unguiculata*
*Sitobion avenae*	*Spiroplasma*	Field	Hebei	Langfang	Female	6	2021	-	*Triticum aestivuml*
*Plutella xylostella*	Microsporidia	Field	Guangdong	Guangzhou	Female	2	2017	-	*Brassica napus*

Note: “-” indicates that the phytophagous insects in the table do not have a host plant.

**Table 2:** Primers used in general polymerase chain reaction (PCR) assays to detect microbes manipulating reproduction.

Organism	Gene	Primers	Sequence (5′–3′)	Annealing (°C)/Product Size (bp)	Positive Controls	References
*Diglyphus wani*	COI	COISF	TAAGATTTTGATTATT(AG)CC(TA)CC	48/~850	-	[31]
		COI2613	ATTGCAAATACTGCACCTAT			[32]
*Encarsia formosa*		LCO1490 HCO2198	TCAACAAATCATAAAGATATTGG TAAACTTCAGGGTGACCAAAAAATCA	52/~800	-	[33]
*Bemisia tabaci*						
*Neochrysocharis formosa*						
*Sitobion avenae*						
*Plutella xylostella*						
*Wolbachia*	*WSP*	81F	TGGTCCAATAAGTGATGAAGAAAC	52/~610	*Encarsia formosa*	[28]
		691R	AAAAATTAAACGCTACTCCA			
*Wolbachia*	*16S*	99F	TTGTAGCCTGCTATGGTATAACT	52/~936		[29]
		994R	GAATAGGTATGATTTTCATGT			
*Cardinium*	*16S*	CLOf	GCGGTGTAAAATGAGCGTG	52/~600	*Bemisia tabaci*	[30]
		CLOr1	ACCTMTTCTTAACTCAAGCCT			
*Arsenophonus*	*23S*	Ars23s-F	CGTTTGATGAATTCATAGTCAAA	52/~650		[34]
		Ars23s-R	GGTCCTCCAGTTAGTGTTACCCAAC			
*Rickettsia*	*16S*	Rb-F	GCTCAGAACGAACGCTATC	56/~900	*Neochrysocharis formosa*	[35]
		Rb-R	GAAGGAAAGCATCTCTGC			
*Spiroplasma*	*16S*	Spixo-F	TTAGGGGCTCAACCCCTAACC	52/~810	*Sitobion avenae*	[36]
		Spixo-R	TCTGGCATTGCCAACTCTC			
Microsporidia	*16S*	V1F	CACCAGGTTGATTCT	57/~1300	*Plutella xylostella*	[37,38]
		1492R	GGTTACCTTGTTACGACTT			
Microsporidia		V1F	CACCAGGTTGATTCT	63/~450		[39]
		530R	CCGCGGCTGCTGGCAC			

**Table 3:** The offspring of 5 generations (P1–P5) produced by thelytokous *Diglyphus wani* under high temperature and antibiotics.

Generations	Tetracycline (20 mg·mL^−1^, n = 10)	Rifampicin (20 mg·mL^−1^, n = 10)	High-Temperature (34 °C, n = 20)
P1	139 Females	260 Females	190 Females
P2	177 Females	235 Females	184 Females
P3	192 Females	302 Females	299 Females
P4	132 Females	229 Females	184 Females
P5	149 Females	207 Females	214 Females
Total	789 Females	1233 Females	1071 Females

## Data Availability

The data presented in this study are available in this article.
